# Unveiling the Micronome of Cassava (*Manihot esculenta* Crantz)

**DOI:** 10.1371/journal.pone.0147251

**Published:** 2016-01-22

**Authors:** Sarah Jane Rogans, Chrissie Rey

**Affiliations:** School of Molecular and Cell Biology, University of the Witwatersrand, Johannesburg, South Africa; University of Balochistan, PAKISTAN

## Abstract

MicroRNAs (miRNAs) are an important class of endogenous non-coding single-stranded small RNAs (21–24 nt in length), which serve as post-transcriptional negative regulators of gene expression in plants. Despite the economic importance of *Manihot esculenta* Crantz (cassava) only 153 putative cassava miRNAs (from multiple germplasm) are available to date in miRBase (Version 21), and identification of a number of miRNAs from the cassava EST database have been limited to comparisons with *Arabidopsis*. In this study, mature sequences of all known plant miRNAs were used as a query for homologous searches against cassava EST and GSS databases, and additional identification of novel and conserved miRNAs were gleaned from next generation sequencing (NGS) of two cassava landraces (T200 from southern Africa and TME3 from West Africa) at three different stages post explant transplantation and acclimatization. EST and GSS derived data revealed 259 and 32 miRNAs in cassava, and one of the miRNA families (miR2118) from previous studies has not been reported in cassava. NGS data collectively displayed expression of 289 conserved miRNAs in leaf tissue, of which 230 had not been reported previously. Of the 289 conserved miRNAs identified in T200 and TME3, 208 were isomiRs. Thirty-nine novel cassava-specific miRNAs of low abundance, belonging to 29 families, were identified. Thirty-eight (98.6%) of the putative new miRNAs identified by NGS have not been previously reported in cassava. Several miRNA targets were identified in T200 and TME3, highlighting differential temporal miRNA expression between the two cassava landraces. This study contributes to the expanding knowledge base of the micronome of this important crop.

## Introduction

MicroRNAs (miRNAs) are an important class of endogenous small RNAs. They are evolutionary conserved, single-stranded, non-coding pieces of RNA that are 21–24 nt in length [1, 2]. MiRNAs serve as post-transcriptional negative regulators of gene expression in plants and animals by negatively regulating their target gene expression at post-transcriptional levels through mRNA cleavage or repression of translation, depending on the complementarity between the miRNAs and their target genes [3–5] MiRNAs regulate a great number of genes involved in plant growth and development, environmental stress response, signal transduction as well as response to pathogen invasion [6].

The biogenesis of mature miRNAs encompasses a co-ordinated interplay of a few cellular proteins in and outside of the nucleus and is a multi-step process. Like their protein-coding counterparts, miRNAs are also transcribed form their own genes, known as *MIR* genes [3, 7]. *MIR* genes are much longer than their mature sequences and range from several tens to more than 1000 nt. Mature miRNAs are produced from a pathway starting with the *MIR* genes being transcribed to the capped and polyadenylated primary miRNA transcripts (pri-miRNA) by the Pol II enzyme [8, 9]. The pri-miRNA forms an imperfect hairpin-like secondary structure, which undergoes cleavage to form a perfect hairpin precursor called precursor miRNA (pre-miRNA) with the aid of Dicer-like enzyme (DCL1), a plant counterpart of the animal Dicer enzyme [2, 10–12]. In the dicing process DCL1 interacts with the pri-miRNA with the aid of DWADLE (DDL), which plays a significant role in recruiting DCL1 to the pri-miRNA [13]. The pre-miRNA is further processed by DCL1 to release the stem portion of the hairpin as a miRNA: miRNA* duplex (miRNA* is the complementary sequence to miRNA on the opposing arm) [2, 10]. Cellular enzymes like HYL1 (HYPONASTIC LEAVES 1), HEN1 (HUA ENHANCER 1) and HST1 (HASTY 1) are obligatory for the maturation of miRNAs. DCL1 associates with its cohort HYL1, a dsRNA binding protein, and SERRATE (SE), a zinc finger protein, to produce mature miRNAs [14, 15]. The processed miRNA duplex is 2’-O methylated and polyuridylated at the 3’-terminal nucleotide by a dsRNA methylase HEN1. The methylation protects miRNAs from degradation [16, 17]. The mature miRNA duplex is then exported out of the nucleus by HST1 an EXPORTIN 5 orthologue in plants [18–20]. Out of the two strands of the mature miRNA duplex (miRNA:miRNA*), the one with the least 5’ end thermodynamic stability will function as a mature miRNA, whereas the other strand (miRNA*) termed the passenger strand is specifically degraded [21]. Finally, the single-stranded mature miRNA is incorporated with AGONAUTE (AGO) proteins to form a ribonucleoprotein complex known as RNA-induced silencing complex, where the regulation of target gene expression occurs [2, 6, 22]. The RISC complex along with the mature miRNA negatively regulates gene expression either by inhibiting translation elongation or triggering messenger RNA (mRNA) degradation depending on the degree of complementarity of the miRNA sequence with its target.

A large number of miRNA families are evolutionary conserved in the plant kingdom, which ranges from mosses and ferns to higher flowering plants [23]. This attribute has been used as a practical indicator for the identification and prediction of miRNAs by homology searches in other species. During recent years the identification and characterisation of miRNA [23] and their target genes from plants has been extensively studied [24, 25, 26]. In the past decade, a large number of miRNAs have been discovered across several plant species; for instance the miRBase database [27] version 21 contained 8524 mature miRNA sequences for 73 plant species. The majority of these miRNAs have been validated using different computational and experimental approaches including deep sequencing, cloning, northern blots and real-time PCR [4, 28, 29].

Comparison of miRNAs in different plant species by expression sequence tags (EST) analysis had shown that some miRNAs were highly evolutionary conserved among species [23]. This provided a powerful strategy for identifying miRNAs in a new plant species. Identification of miRNAs using EST analysis has two significant advantages [30]: there is no specialized software required and it can be used to identify miRNAs in any species if they have previously registered EST sequences. Since ESTs are derived from transcribed sequences, EST analysis also provides direct evidence for miRNA expression. In view of these advantages, EST analysis had been used to identify conserved miRNAs in several plants including *Brassica napus* [31], *Medicago trunculata* [32], *Lycopersicon esculentum* [33], *Glycine max* [34], *Nicotiana tabacum* [35], and *Solanum tuberosum* [36]. In addition, an *in silico* search of miRNAs in public databases and a bioinformatics approach can greatly assist to identify miRNAs in several plants [37] especially those whose complete genome sequences are unavailable. It has also been suggested that most of the miRNAs predicted from EST analysis can also be identified by high throughput deep sequencing [38].

There are several miRNAs considered to be recently evolved that show species-specificity and are often expressed at lower levels. Many are tissue specific, and are expressed at certain stages in development or under specific growth conditions, relative to the highly conserved group of miRNAs [39, 40]. Next generation sequencing (NGS) technology has great promise to generate an accurate and comprehensive picture of the small RNA transcriptome in different plants, tissues, and at different developmental stages. Using deep sequencing, both species-specific (novel) and conserved miRNAs have been identified in diverse plant species such as *Arabidopsis* [40, 41], tomato [42, 43], cucumber [44], maize [45], peanuts [46], pepper [47] and rice [48]. However, homology-based searches in databases are not sufficient for identifying miRNAs; therefore other additional criteria have been set for distinguishing miRNAs from other types of small RNAs. Predicting the secondary structure of the pre-miRNA and calculating the free energy are necessary for reducing the number of false positive identified miRNAs [1, 49–51].

Cassava (*Manihot esculenta* Crantz) is a crop widely grown as a staple food and along with maize, sugarcane and rice is a major source of energy for more than 700 million people in most tropical countries including sub-Saharan Africa [52]. Apart from its traditional role as a food crop, there is a growing demand for cassava starch in a diverse set of industries such as animal feed, paper, textile and adhesive as well as an alternative energy resource [53]. Despite the economic importance of cassava and the potential contribution of miRNAs to cassava improvement, molecular genetic information regarding cassava miRNAs remains sparse. Only recently, 153 conserved miRNAs were made available in miRBase (Version 21 for cassava [27], however other well-studied plant species such as *Arabidopsis thaliana*, *Glycine max*, *Populus trichocarpa* and *Oryza sativa* have 427, 639, 401 and 713 reported miRNAs in miRBase, respectively [27]. The miRNAs that are available for cassava on miRBase were obtained by Patanun *et al*., [54] using a computational prediction method by using homology search based on miRNA conservation among different plant species. In addition Perez-Quintero *et al* [55] analysed small RNA libraries from cassava tissues infected and uninfected with *Xanthomonas axonopodis*, and Zeng *et al* [56] studied conserved miRNAs in the *Euphorbiaceae* family. More recently, Ballen-Taborda *et al*. [57] and Xia *et al*. [58] both studied cassava miRNAs expressed under abiotic stress conditions.

The identification of a more comprehensive set of miRNAs in cassava is a critical step to facilitate our understanding of regulatory mechanisms or networks, in particular responses to viral pathogens, of particular interest in our laboratory. In this study we employed a combinatorial approach of publically available cassava EST and GSS data in NCBI, and next-generation sequencing-derived miRNA data collected at 8, 10 and 15 weeks post-planting from two cassava landraces, T200 and TME3, to systematically identify conserved and novel miRNAs in cassava. Our findings revealed 259, 32 and 289 conserved miRNAs using the EST, GSS and NGS data respectively and 39 novel cassava-specific miRNAs of low abundance, belonging to 29 families. In order to understand the function of the newly identified conserved and novel miRNAs in cassava, the targets of these miRNAs were also identified. The knowledge gained from this study contributes to the cassava miRNA database and micronome of this important crop, and unveils differences between landraces, which will be beneficial in the long term in linking gene regulation, gene targets and germplasm traits.

## Results and Discussion

### Small RNA sequencing analysis

In order to identify novel and conserved miRNAs in two cassava landraces, six small RNA-enriched libraries were generated from cassava leaves that were collected from two cassava landraces, T200 and TME3, at 8, 10 and 15 weeks after transferring plantlets from tissue culture to Jiffy^®^ pellets using the Illumina HiSeq2000 system. The small RNA sequencing yielded a total of 64 827 692 raw reads for the six libraries (Table 1).

**Table 1:** Summary of small RNA sequencing data analysis.

	T200 8 weeks	T200 10 weeks	T200 15 weeks	TME3 8 weeks	TME3 10 weeks	TME3 15 weeks
**Raw**	6 774 098	14 687 734	23 163 380	2 914 650	4 589 392	12 692 438
**Adapter-trimmed Reads**	5 921 995	12 914 563	20 603 045	2 668 525	3 625 654	11 414 040
**15–50 nt**	4 692 091	10 248 438	15 487 598	2 209 332	2 788 194	9 196 014
**18–26 nt**	1 436 570	4 381 089	6 935 189	634 217	479 048	2 435 899
**Normalised 18–26 nt**	212 061.83	298 282.158	299 403.153	217 596.28	104 381.58	191 917.35
Total significant Rfam matching sequences*	23 273	34 405	65 997	23 005	15 438	48 811
**rRNA**	9 032	11 170	22 841	11 129	6 764	15 965
**tRNA**	5 106	9 873	14 883	2 913	2 604	5 634
**snoRNA**	2 387	3 190	5 641	1 797	1 286	5 451
**Total mapped to miRBase V.20**	42 339	57 945	221 841	33 208	30 539	88 200
**Exact Matches (Before Hairpin Filtering)**	5 150	10 779	63 594	4 294	1 909	9 784
**isomiRs (Before Hairpin Filtering)**	23 542	28 269	101 274	17 729	22 419	36 304

* Reads that had an E-value of <0.05

After removing low-quality sequences, adapters, and small sequences (<15 nt), 68.8% (44 621 667 reads) of the raw reads remained. A final filtering step, to obtain the sequences that have sizes between 18 and 26 nt, yielded a total 16 302 012 reads for the six libraries (Table 1). The 18–26 nt libraries were normalized per million read counts in order to compare sRNA abundance data. The next filtering step involved the removal of non-coding RNAs such as ribosomal RNA (rRNA), transfer RNA (tRNA) and small nuclear RNA (snoRNA). This filtering step was performed by conducting a BLASTn search of the small RNA libraries against the RNA families database Rfam [59] (Table 1). Only sequences with perfect matches and an E-value <0.06 were removed from the libraries.

**Fig 1:**
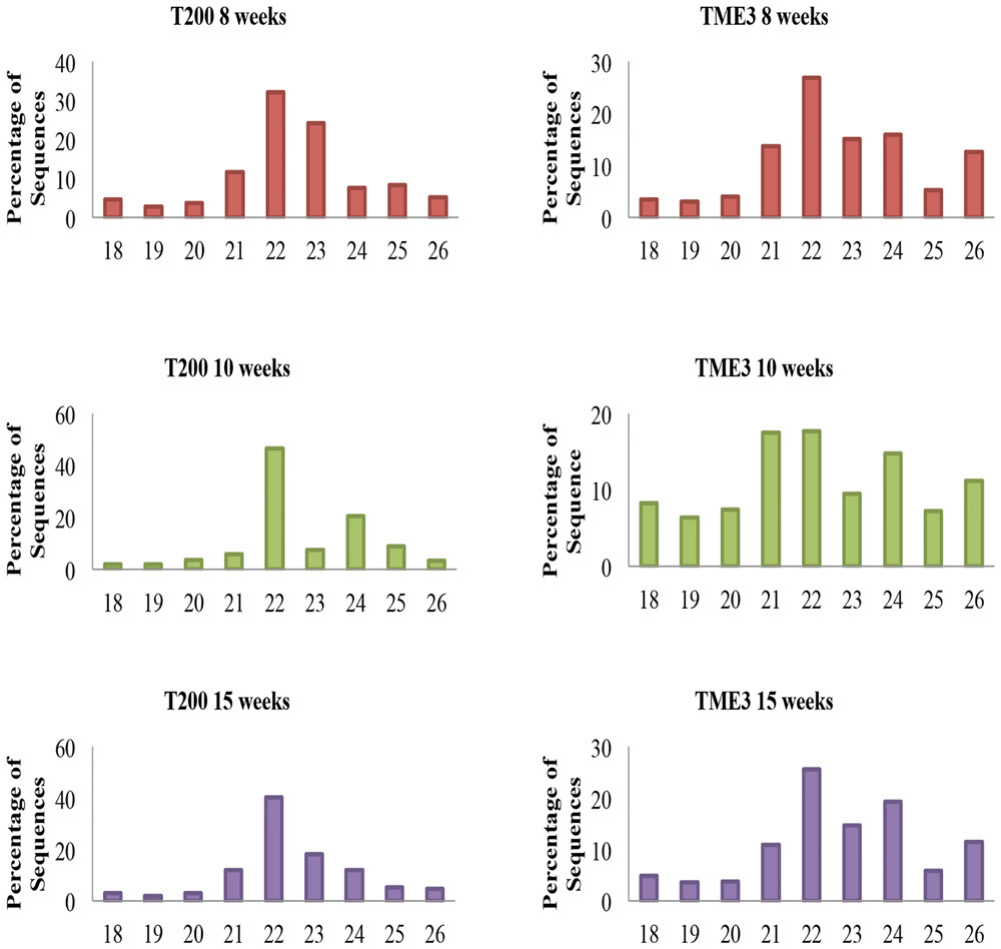
Sequence length distribution of cassava small RNAs from T200 and TME3 landraces. Percentage of sequences of 18–26 nt length for each of the six sequenced libraries. The majority of the generated reads were 21 to 24 nt in length.

The size distribution analysis of small RNA (sRNAs) sequences exhibited a similar pattern of length distribution in all libraries. The small RNA length distribution (18–26 nt) of each library showed that the most abundant and diverse species were those 21–24 nt in length (Fig 1), which is typical of Dicer-derived products [60]. In all six libraries, while the 21 nt size class is characteristic of authentic miRNAs [60], it was most intriguing to note that the 22 nt class was the most abundant, followed by the 23 nt class for T200 at 8 weeks (24.2%) and T200 at 15 weeks (18.1%). The 22 nt miRNA or miRNA* length is important for triggering secondary siRNA biogenesis [6, 61]. The 22-nt miRNAs or miRNA* are often generated from asymmetric miRNA precursors. The asymmetric miRNA precursors affect the structure of the miRNA/miRNA* duplex, allowing RISC to recruit the RDR6 and SGS3 to trigger the formation of the secondary siRNA [62, 63]. The 21 nt class sRNAs was under-represented in T200 at all 3 timepoints compared to the 22 nt sRNAs, and for TME3, the percentage of 21 nt sRNAs only comprised 17% and 11.5% of the total number of sRNAs in the 10 and 15 week library, respectively. Studies in grapevine [64], wheat [65]; Chinese yew [66] and potato [67] also found the 23 nt class to be one of the more abundant size classes in their sRNA libraries. The 24 nt sRNA class was less abundant: for the TME3 8 weeks (15.9%), T200 10 weeks (20.5%) and TME3 15 weeks (19.3%), The presence of the 24 nt small RNAs in our libraries may indicate the complexity of the cassava genome as they are mainly siRNAs that are associated with repeats and heterochromatic modifications [61].

### Identification of conserved miRNAs in cassava

#### Identification of potential conserved cassava miRNAs from EST and GSS databases

**Fig 2:**
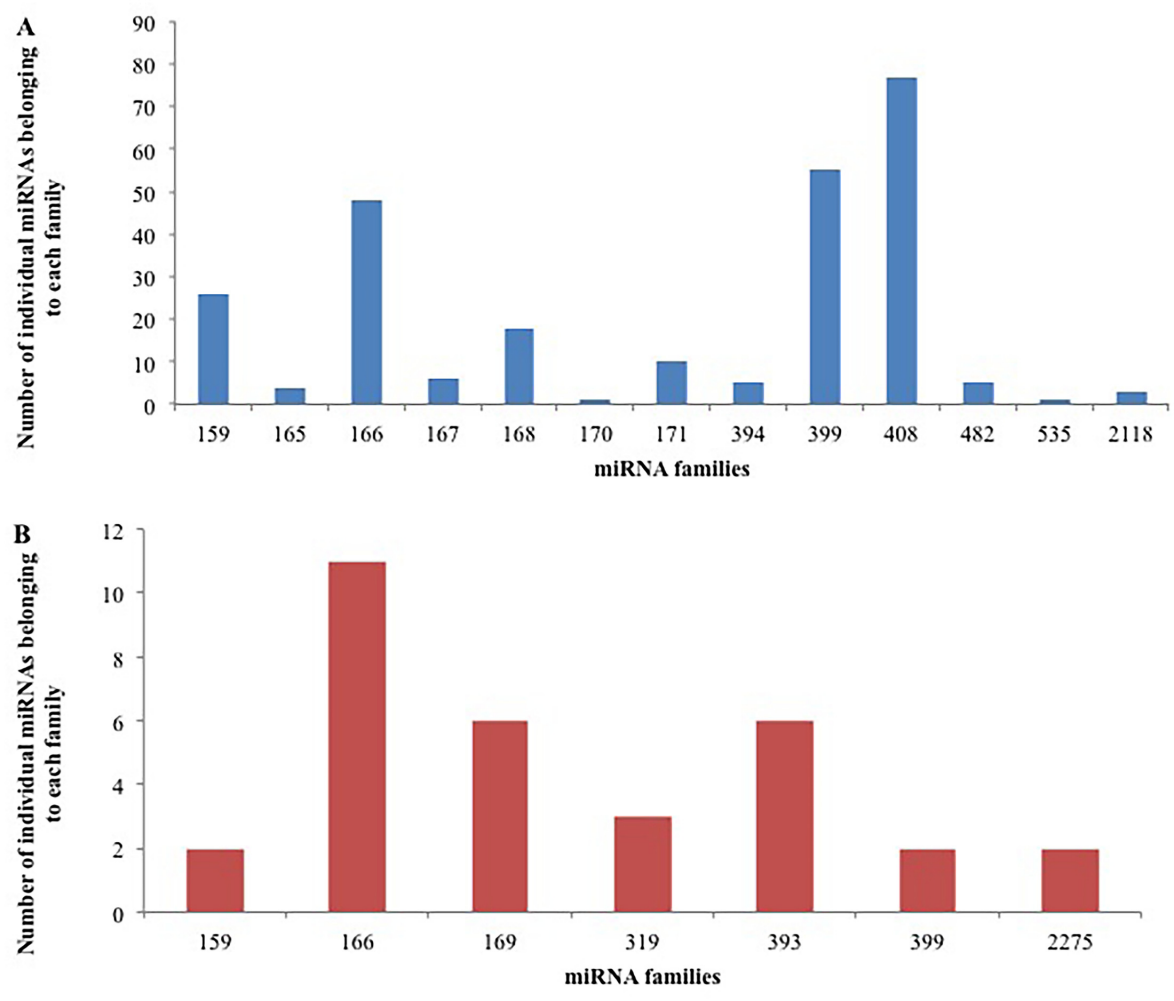
The number of individual miRNAs belonging to each miRNA family identified in cassava using (A) EST database and (B) GSS database.

In order to profile and characterize the potential miRNAs in cassava, a comparative genomic approach along with computational and bioinformatics tools was used. In this study, 259 miRNAs in cassava from EST data (S1 Table) and 32 miRNAs from GSS data (S2 Table) were identified. One of the miRNA families, miR2118 identified in this study using the EST database has not been reported in cassava in previous studies [55–58, 68]. The 259 putative cassava miRNAs identified using the EST database belong to 13 families. The largest family was miR408 with 84 individual members and the smallest families were miR170 and miR353 with 1 member each (Fig 2A). The 32 miRNAs identified using the GSS database belong to 7 families. The miR166 family was the largest family with 11 members, while the miR399, miR2275 and miR159 families were the smallest only containing 2 members each (Fig 2B). Also, there were 3 miRNA families that were identified in both EST and GSS databases, miR159, miR166 and miR399. Three miRNAs belonging to the miR166 family were also common to both the EST and GSS databases.

#### Identification of potential conserved cassava miRNAs from high throughput next generation sequencing (NGS) data

**Fig 3:**
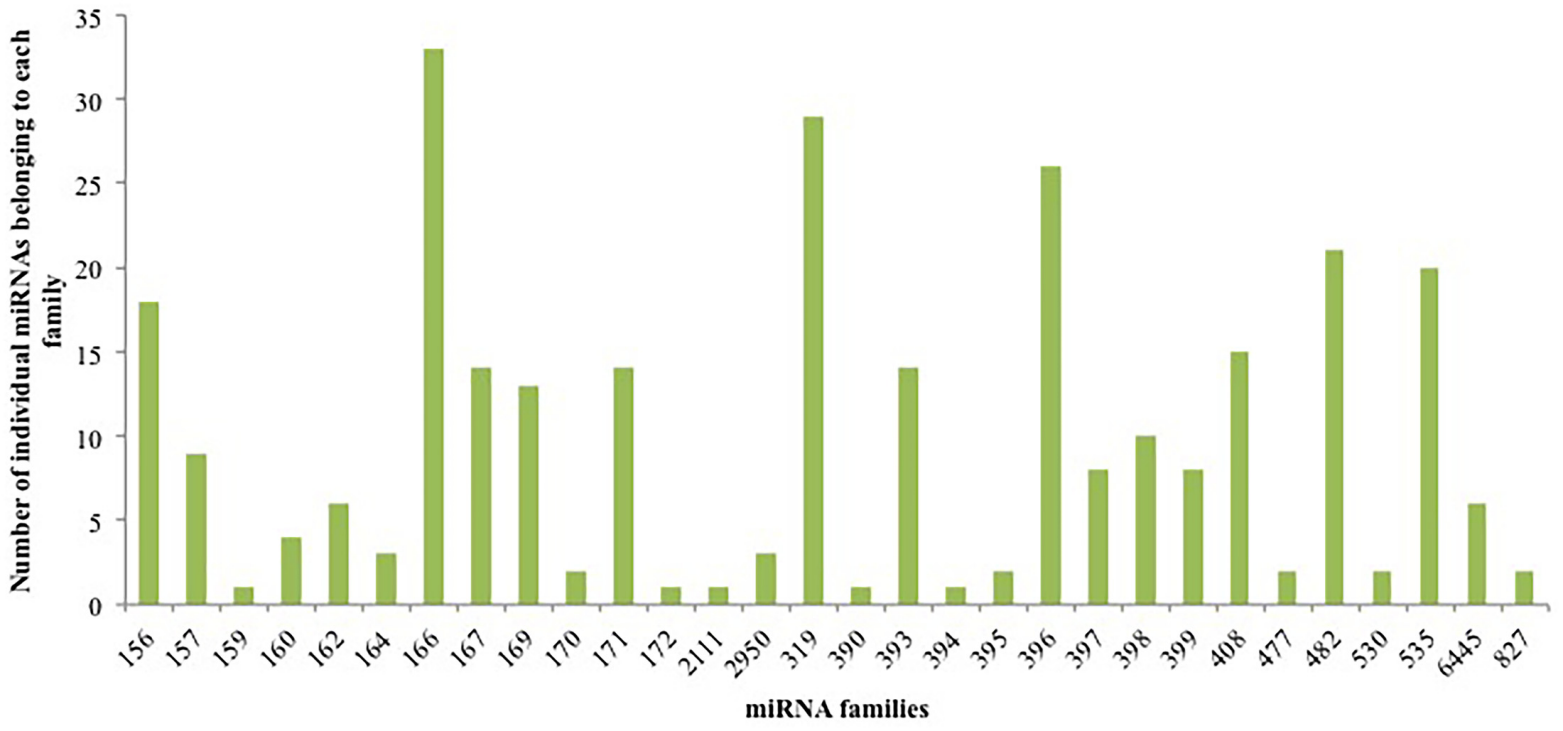
The number of conserved individual miRNAs belonging to each miRNA family identified in cassava T200 and TME3 from deep sequencing data.

In order to confirm EST and GSS data-derived miRNA results, and identify additional conserved miRNAs in cassava T200 and TME3 landraces, unique sRNA sequences from NGS data at 8, 10 and 15 weeks after transfer from tissue culture to Jiffy^®^ pellets were aligned against the known plant miRNAs deposited in miRBase (Version 21) with a maximum of three mismatches in CLC genomics workbench. A total of 289 potential conserved cassava miRNA sequences belonging to 30 miRNA families were identified from both landraces and developmental stages collectively in this study (S3 Table). Of the 30 miRNA families, the miR166 family was the largest with 33 members. The four families, miR319, miR396, miR482, and miR535 were found to contain 29, 26, 21, and 20 families, respectively. The remaining 25 families contained less than 20 members with 18 of the families containing less than 10 members (Fig 3). It has been previously suggested that most of the miRNAs predicted from EST analysis can be recovered by high throughput NGS [38]. In this study 99 (38.2%) of the miRNAs that were identified using the EST database were also identified in the NGS data and are highlighted in green in S1 Table. We were also able to identify 9 (28.1%) of the miRNAs that were identified using the GSS database using the NGS data (highlighted in green in S2 Table).

### Characterization of the newly identified conserved cassava miRNAs

#### EST and GSS data

**Fig 4:**
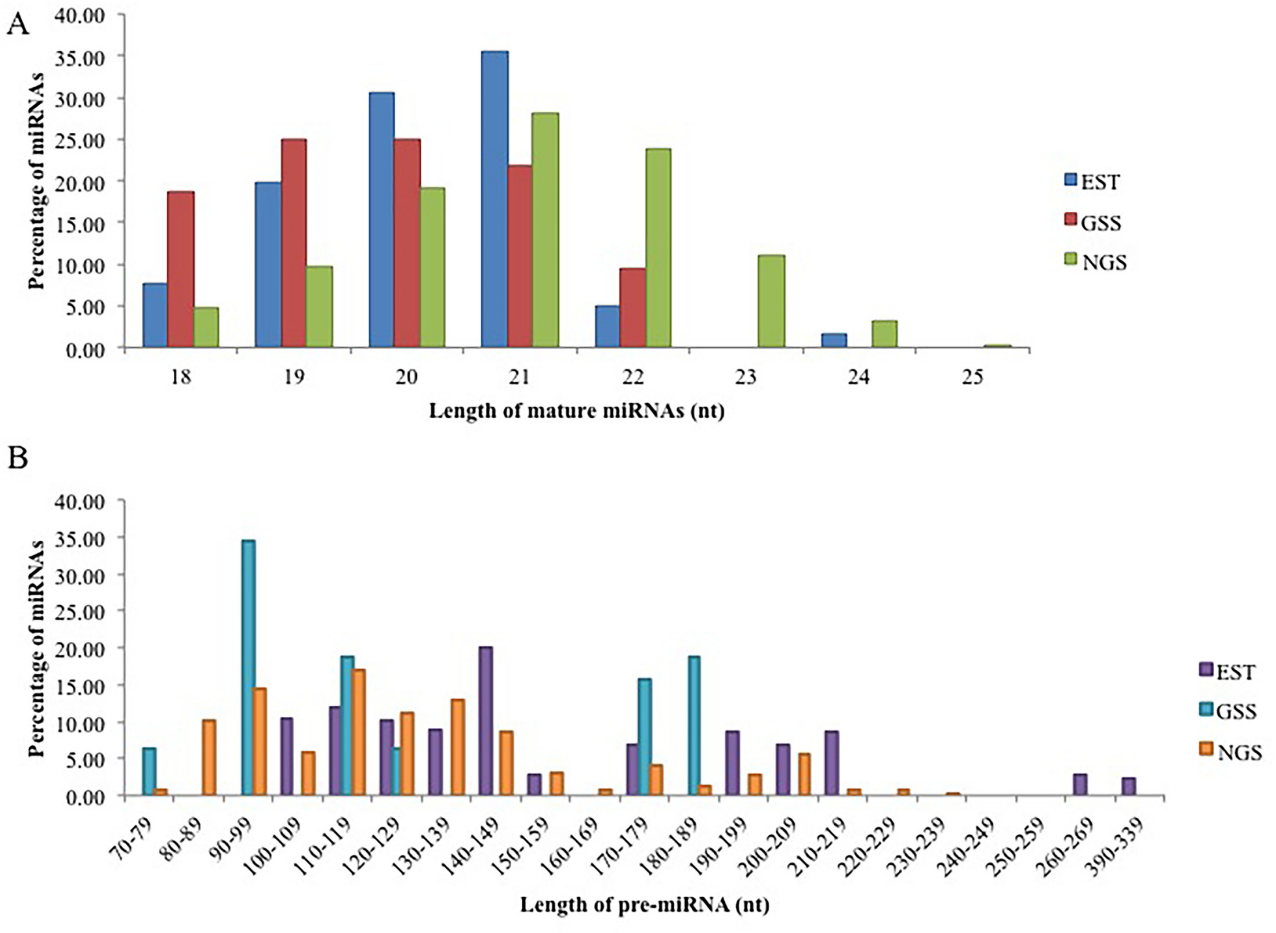
(A) Size distribution of the conserved mature miRNAs and (B) pre-miRNAs identified using the EST database, GSS database and NGS data.

Characterization of putative candidate miRNAs is a crucial step for their validation as it distinguishes miRNAs from other small RNAs (i.e. tRNAs, rRNAs and mRNAs), as reported earlier [35, 69]. The newly identified potential cassava miRNAs characterized from EST and GSS databases, using accepted criteria/characteristics are summarized in S1 and S2 Tables. The mature miRNA sequences identified from EST database ranged from 18 to 24 nt. The majority (37%) of the miRNAs are 20 nt in length, followed by 21 nt (28%), 19 nt (19%), 18 nt (11%), 22 nt (4%), and 24 nt (1%) (Fig 4A). The mature miRNAs identified from the GSS database ranged from 18 to 22nt. The majority of the miRNAs either had a length of 19 nt (25%) or 20 nt (25%), followed by 21 nt (22%), 18 nt (19%) and 22 nt (9%) (Fig 4A). These findings are in agreement with previously reported studies in other plants species [24, 34, 35, 45, 69, 70]. The lengths of the potential precursor miRNAs varied from 100 nt to 775 nt for EST-derived (Fig 4B) data and 76 nt to 187 nt for the GSS-derived data (Fig 4B). These results are similar to previous reports in *Arabidopsis*, potato, and rice [5, 34, 71]. The average A/U% for the pre-miRNAs identified from EST database was 56% and ranged from 42% to 63% and the pre-miRNAs identified from the GSS database also had an average A/U% of 56% and ranged from 47% to 64%. These results are in agreement with the criteria described by Zhang et al [49], as the A/U% of a potential pre-miRNA should be with 30–70%. In the stem-loop hairpin pre-miRNAs sequences, 66% of the mature miRNAs identified from the EST database were located on the 3’ arm, while 34% were located at the 5’ arm. The majority (69%) of the mature miRNAs identified from the GSS database were also located at the 3’ arm, while 31% were located at the 5’ arm.

The determination of a hairpin-loop secondary structure of a potential miRNA is not enough for distinguishing miRNAs from other types of non-coding RNAs [49, 50]. The minimal folding free energy (MFE) is an important criterion to determine stability of the perfect or near-perfect secondary hairpin structure of pre-miRNAs. The more negative the value of MFE, the higher the thermodynamic stability is of the secondary structure of the precursor sequence. The MFE of the pre-miRNAs identified from the EST database ranged from -39.9 kcal/mol to -131.3 kcal/mol. The MFE of the pre-miRNAs identified from the GSS database ranged from -26.4 kcal/mol to -95.2 kcal/mol. The minimal folding free index (MFEI) is an important criterion for distinguishing miRNAs from other RNAs. Previous research has suggested that a sequence is more likely to be a potential miRNA if the pre-miRNA had a MFEI more negative than -0.85 kcal/mol [72]. The putative cassava pre-miRNAs identified from the EST database MFEIs ranged from -0.847 kcal/mol to -1.207 kcal/mol. The pre-miRNAs identified from the GSS database MFEIs ranged from -0.964 kcal/mol to– 1.183 kcal/mol. Therefore the cassava pre-miRNAs identified in this study had more negative MFEIs than other types of RNAs: tRNA (0.64); rRNAs (0.59); mRNAs (0.65) [72], lending support for their identification as pre-miRNAs.

#### Next Generation Sequencing Data

A summary of the important characteristics of the miRNAs identified from the NGS data from T200 and TME3 landraces can be found in S3 Table. The identified potential cassava mature miRNA sequences ranged in size between 18–25 nt in length. Most of the mature miRNAs were 21 nt in length (28.02%) followed by 22 nt (23.87%), 20 nt (19.03%), 23 nt (7.9%), 19 (9.68%), 18 nt (4.84%), 24 nt (3.11%), and 25 nt (0.34%) (Fig 4A). Of the 289 identified cassava miRNAs, 170 (58.82%) were found to be located on the 3’ arm of the hairpin secondary structure, while the remaining 119 (41.17%) were located on the 5’ arm. It was also found that miRNAs belonging to the same family miRNA family were not required to be located on the same arm of the pre-miRNA. Most of the identified cassava mature miRNA sequences began with the base uracil (U) (57.78%), which was consistent with previously reported results in other plants [28, 73], due to the high affinity of AGO proteins to bind with U base in the 5’ terminus of mature miRNAs sequences [74].

The identified cassava miRNA precursor sequences ranged from 70–233 nt in length with an average length of ±127 nt (Fig 4B). The nt composition of these precursor sequences had an average G+C% of 44.35% and A +U% of 55.64% and A +U% ranged from 34.22%–73.34%, which is consistent with the miRNA secondary structure filtering criteria by Zhang *et al*. [49]. The average MFE of the cassava pre-miRNAs was -58.08 kcal/mol. In this study the determined MFEI values of the cassava pre-miRNAs ranged from -0.84 to -1.70 kcal/mol, with an average of -1.03kcal/mol, strongly supporting the validity of these predicted pre-miRNAs in cassava.

### RT-PCR validation of data

**Fig 5:**
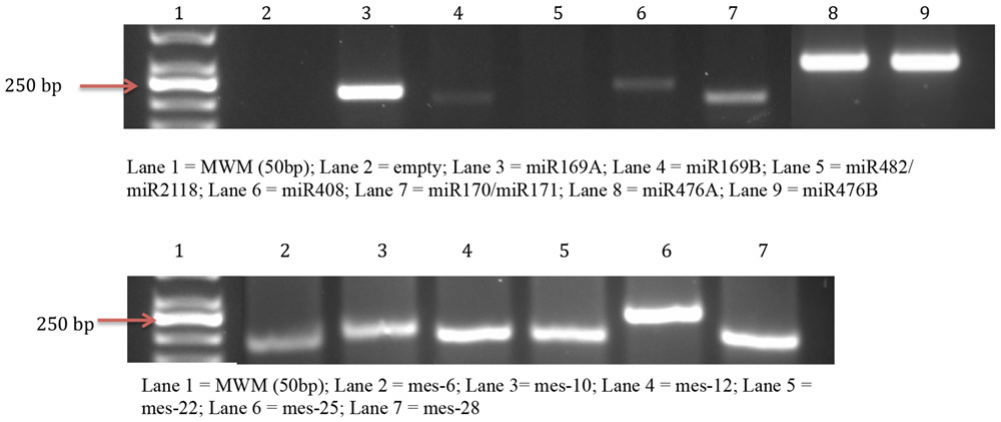
Cassava miRNA RT-PCR expressional validation of the identified (A) conserved cassava miRNAs and (B) novel cassava specific miRNAs. The product of each sample was separated on a 2% agarose gel. (A) Lane 1 = MWM (50bp); Lane 2 = empty; Lane 3 = miR169A; Lane 4 = miR169B; Lane 5 = miR482/miR2118; Lane 6 = miR408; Lane 7 = miR170/miR171; Lane 8 = miR476A; Lane 9 = miR476B. (B) Lane 1 = MWM (50bp); Lane 2 = mes-6; Lane 3 = mes-10; Lane 4 = mes-12; Lane 5 = mes-22; Lane 6 = mes-25; Lane 7 = mes-28.

Seven identified conserved miRNAs: miR169, miR170/171, miR408, miR476 and miR482/2118 were selected for RT-PCR validation studies. All the miRNAs were experimentally validated except for miR482/miR2118 (Fig 5A). This could be due to these miRNAs being present at very low levels in cassava or they could be tissue or developmental stage specific. The experimental validation of these miRNAs provides additional support for the computationally identified miRNAs.

### Identification of miRNA isoforms

Small non-coding RNAs such as miRNAs were initially thought to have a specific sequence of a defined length. Identification of more miRNAs from different species has revealed that there is variation in pre-miRNA processing. A single miRNA locus can give rise to multiple distinct isomiRs that differ in their length and sequence composition [75]. In the conventional plant miRNA biogenesis pathway, the 5’ and 3’ ends are specified by consecutive cleavage events of the primary transcript by the ribonuclease Dicer-like 1 (DCL) [2]. In this study in cassava, of the 289 conserved miRNAs identified in T200 and TME3 using NGS data, 208 were isomiRs. These 208 isomiRs belonged to 27 families. The most frequently observed type of isomiR in both plants and animals is the 3’ isomiRs, in terms of both number of miRNAs displaying these variations and their overall abundance [76,77]. Seventy nine (±38%) out of the 208 isomiRs in this study were also found to be 3’ isomiRs.

IsomiRs are categorised into three main classes: 5’ isomiRs, 3’ isomiRs, and polymorphic isomiRs, with 5’ and 3’ isomiRs subclassified into templated or non-templated modifications [76]. Heterogeneity in length can arise from the imprecise cleavage by DCL, in which case the miRNA sequences will match the parent gene but will vary in length, a situation referred to as ‘templated’. Length heterogeneity can also arise by exonucleases ‘nibbling’ off the end, which produces a shorter templated product, which is referred to as sub-templated. They can also arise from post-transcriptional addition of one or more bases, which is referred to as super. The addition of these bases can result in the end matching the parent gene, templated, or the end may not match the parent gene and is known at non-templated. Polymorphic isomiRs harbour different internal nucleotide sequences, but these are relatively rare [76]. Forty-three of the 3’ isomiRs from T200 and TME3 were classified as sub-templated and 36 were classified as super-templated. The super templated 3’ isomiRs were further divided into 33 super templated and 3 super non-templated. The second largest class was the 5’ isomiRs. There were 45 (±22%) 5’ isomiRs divided into 16 sub templated and 29 super templated. Twelve polymorphic isomiRs were identified with either 1 or 2 nucleotides involved in a mismatch between the isomiR and the reference miRNA. There were also 52 identified isomiRs that had changes in length at both the 5’ and 3’ ends but contained no sequence differences between itself and the reference miRNA. Twenty isomiRs contained both length differences at both ends as well as polymorphic changes.

IsomiRs were also identified using the EST and GSS databases. For the miRNAs identified from the EST database, 101 of the potential cassava miRNAs were completely identical to their reference miRNA, while 158 were variants of their reference miRNA and are isomiRs (bolded in S1 Table). The largest class of isomiRs was the 3’ sub templated (31.6%), followed by the polymorphic class with (24.1%), the 5’ sub templated class (22.8%), the 3’ sub polymorphic class (12%), 5’ sub polymorphic class (7%) and the 5’ and 3’ sub templated class (2.5%). For the miRNAs identified from the GSS database, 12 of the 32 were identical to their reference miRNAs and 10 were isomiRs (bolded in S2 Table). Again the largest class was the 3’ sub templated class (60%) followed by the 5’ sub templated class (20%), 3’ sub polymorphic class (10%) and the 5’ and 3’ sub templated and polymorphic classes with 5% each. No super isomiRs were identified in the EST and GSS databases. The main processing steps in the canonical miRNA biogenesis pathway are the sequential cleavage steps catalysed by the Dicer endonucleases [78], which are a source of templated miRNA variation. However, the fact that variability is most commonly associated with the 3’ end suggests that other processing activities contribute to the distribution pattern. Insights form Argonaute (AGO) crystallographic studies for example, indicating that the 5’ ends of the microRNAs are buried within the MID domain, whereas the 3’ ends extend from the PAZ domain and are therefore available to exonucleolytic attack [79], causing shortening. Also, most nucleotidyl transferases that catalyse the addition of nucleotides are 5’– 3’ polymerases, thereby causing an abundance of nontemplated nucleotide extensions at 3’ rather than 5’ ends [80].

### Identification of novel miRNAs

**Fig 6:**
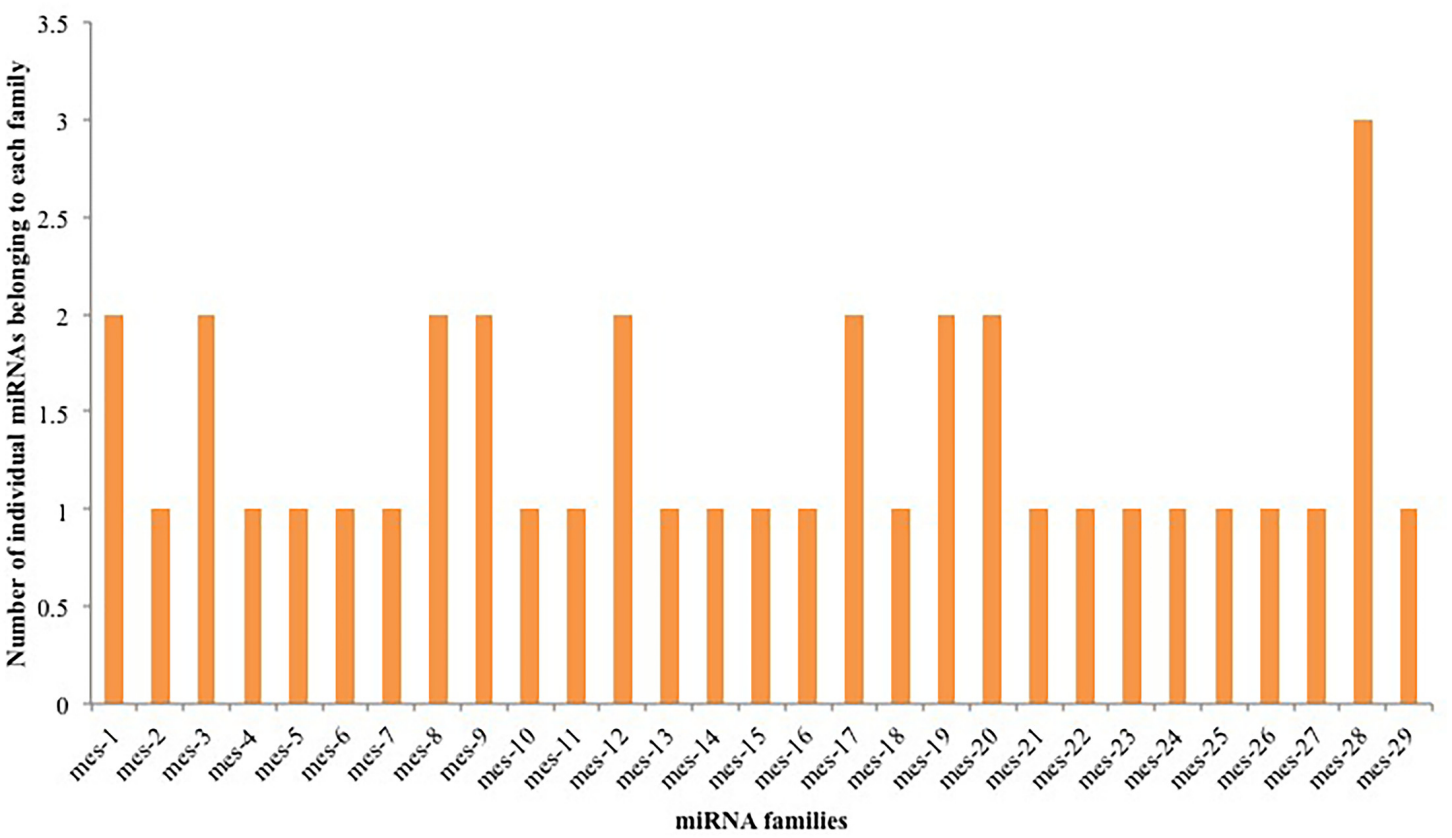
The number of individual cassava-specific miRNAs belonging to each novel miRNA family identified in cassava using NGS data.

**Fig 7:**
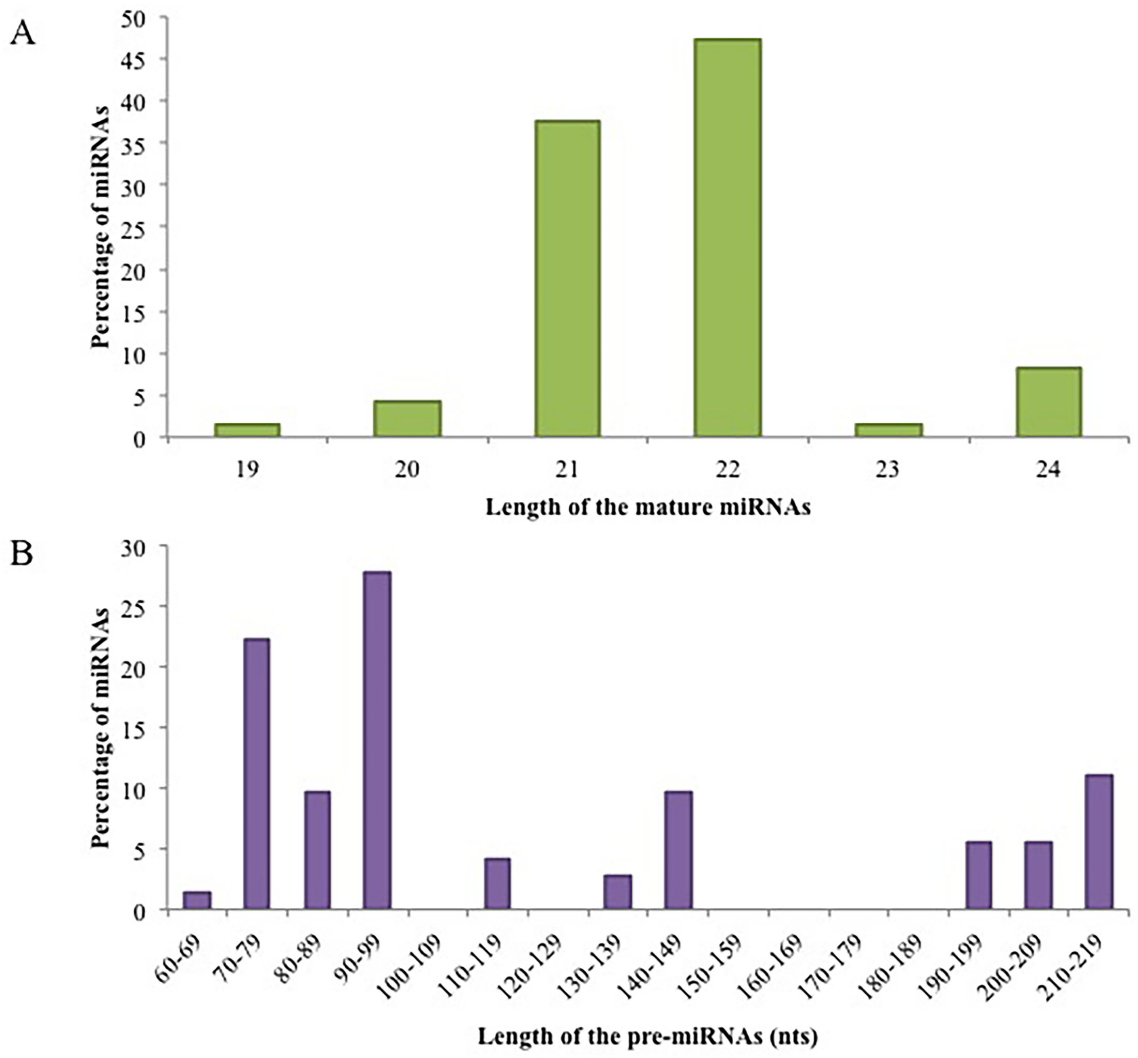
Size distribution of the (A) novel mature miRNAs and (B) pre-miRNAs identified in the deep-sequencing data.

Using the miRCat program in the UEA small RNA workbench [81], 39 novel cassava-specific miRNAs belonging to 29 families were identified and were named mes-1 to mes-29 (Table 2; S4 Table). The largest family was mes-28 containing 3 members. Seven families contained 2 members and the remaining 22 families only contained a single member (Fig 6). The low abundance (< 3) of miRNAs in each family (Fig 6) provides strong evidence that these are cassava-specific miRNAs. Chavez Montes *et al*. [82] have suggested from their study of miRNAs in vascular plants that the majority (92 to 99%) of species-specific new miRNAs occur in low abundance of less than 10 RPM. The majority of the identified potential novel miRNAs in this study were 22 nt in length (47%) followed by 21 nt (38%), 24 nt (9%), 20 nt (4%), and 19 nt (2%) respectively (Fig 7A). Most of the novel mature miRNA sequences began with the base uracil (U), which is consistent with previously reported results in other plants [73], due to the high affinity of AGO proteins to bind with U base in the 5’ terminus of mature miRNAs sequences [74]. The average length distribution of the predicted novel miRNA precursor sequences was 120.5 nt (Fig 7B). The mes-16 family exhibited the shortest precursor length of 67 nt, whereas the mes-7 and mes-8 family members exhibited the longest precursor length of 212 nt. The nt composition of the newly identified potential cassava novel miRNA precursor sequences had an average A+U content of 55.1% and G+C content of 44.8%. The average minimal folding free energy (MFE) of the potential cassava novel pre-miRNAs was -58.9 kcal/mol. In this study, the MFEI for the novel miRNA precursors ranged from -0.89 to -1.56 kcal/mol with an average of -1.12 kcal/mol, which agrees with the important rule that plant miRNA precursors should have a MFEI more negative than -0.85 kcal/mol.

**Table 2:** Novel miRNA families identified in cassava T200 and TME3 and at 3 stages of development post acclimatization from tissue culture explants.

*Novel miRNA families identified		**No. novel miRNA families identified		
T200	TME3	at 3 developmental stages		
		8 wks	10 wks	15 wks
*mes-1*	*mes-2*	mes-8	mes-7	mes-1
mes-3	mes-3	*mes-12*	*mes-12*	mes-2
mes-5	*mes-4*	*mes-13*	*mes-13*	mes-3
*mes-6*	mes-5	mes-20	mes-14	mes-4
mes-8	*mes-7*	*mes-22*	mes-15	mes-5
mes-12	mes-8	mes-24	mes-17	mes-6
*mes-13*	*mes-9*	*mes-25*	*mes-22*	mes-9
*mes-14*	*mes-10*	*mes-28*	*mes-25*	mes-10
*mes-15*	*mes-11*	mes-29	*mes-28*	mes-11
mes-17	mes-12			mes-12
mes-19	*mes-16*			mes-13
*mes-20*	mes-17			mes-16
mes-22	*mes-18*			mes-17
*mes-23*	mes-19			mes-18
mes-25	*mes-21*			mes-19
*mes-27*	mes-22			mes-20
mes-28	*mes-24*			mes-21
	mes-25			*mes-22*
	*mes-26*			mes-24
	mes-28			*mes-25*
	*mes-29*			mes-26
				mes-27
				*mes-28*

*miRNA families that are in italics are the miRNA families that are specific to either cassava landrace T200 or TME3

**miRNA families that are in italics are the miRNA families that are common to both cassava landraces and all three developmental stages

It was observed that different miRNAs belonging to the same miRNA family were produced from the same scaffold and hairpin. Mes-17a and mes-17b were both produced from the same hairpin that is located in scaffold 00631. This was also observed for mes-19a and mes-19b that were produced from the same hairpin located in scaffold 09876. It was also found that miRNAs belonging to different miRNA families could be produced from the same hairpin. Mes-20a, mes-20b and mes-21 were all produced from the same hairpin located in scaffold 01701. However, mes-6, mes-22 and mes-29 were produced from different hairpins but all the hairpins were located in scaffold 03581. This was also observed for mes-8 and mes-9 that had two cases of members being derived from different hairpins that were located in the same scaffold, scaffold 03429 and scaffold 06557 (S4 Table).

Not all the novel miRNAs identified in this study were identified in both cassava landraces at all three growth stages post transfer of explants from tissue culture (8, 10 and 15 weeks). Only 17 miRNAs were identified in the T200 landrace and 8 were T200 specific. In TME3 21 of the novel miRNA families were identified and 12 of them were TME3 specific. These results are summarized in Table 2. In both the 8 and 10 weeks samples, 12 novel miRNA families were identified. The 15 weeks samples had 23 of the novel miRNA families being identified. Only 5 of the identified novel miRNAs were reported for both cassava landraces and in all three developmental stages post explant establishment, namely mes-12, mes-13, mes-22, mes-25 and mes-28 (Table 2). Notwithstanding that different spatial expression patterns of miRNAs may occur, T200 and TME3 were cultivated under the same growth conditions in the growth chamber, and differences in expression between the landraces are therefore likely a consequence of temporal factors and genotype.

The newly identified novel cassava miRNAs were experimentally validated using RT-PCR. The randomly selected 6 novel miRNAs: mes-6, mes-10, mes-12, mes-22, mes-25 and mes-28 were used for the RT-PCR validation studies. All 6 novel miRNAs were experimentally validated (Fig 5B). This experimental validation of these miRNAs strengthens the expressed nature for computationally identified miRNAs.

### Comparison of current study with previous studies involving cassava miRNA identification

The first homology-based comparative genomics cassava study was by Amiteye *et al*. [68]. They used 212 previously reported *Arabidopsis thaliana* mature miRNA and precursor sequences that were available in miRBase Version 14 as a reference for a BLASTn search against the publically available cassava EST database at NCBI (Table 3). This approach resulted in the identification of 35 individual miRNAs belonging to 17 families and their corresponding target genes in cassava that were also conserved in other plant species. However, the ESTs representing 7 of these miRNA families produced foldback structures that showed more than 3 nts not involved in canonical base pairing within a loop or bulge in the mature miRNA:miRNA* duplex. These miRNA families should not have been considered as true miRNAs as they do not follow all the miRNA identification criteria by Ambros *et al*. [1] and Zhang *et al*. [72]. These families are in italics in Table 4. Also, there were also 3 miRNA families that had a MFEI less negative than -0.85 kcal/mol. Plant pre-miRNAs should have a MFEI more negative than -0.85 kcal/mol [72] and these 3 families should also not be considered as true miRNAs (bolded in Table 4).

**Table 3:** Comparison of four previous studies and this study on miRNA identification in cassava.

	Amiteye *et al*., 2010	Perez-Quintero *et al*., 2012	Patanan *et al*., 2013	Ballen-Taborda *et al*., 2013	Xie *et al*., 2014	This study	T200 & TME3 This Study
**Method**	EST homology based	Deep-sequencing (Illumina platform)	Genome-wide homology based search	Deep-sequencing (Illumina platform)	Deep-sequencing (Illumina platform)	EST and GSS homology Based search	Deep-sequencing (Illumina platform)
**Conditions**	N/A	Infected and unifected with *Xanthamonas axonopodis pv*.	N/A	Heat and drought-like conditions	Severe and moderate chilling stresses	N/A	Three developmental stages
**Cassava landrace/cultivar**	N/A	MBRA685	AM5602	TAI16	SC124	N/A	T200 and TME3
**Part of plant**	N/A	Leaves and stem	N/A	Leaves	Leaves and Roots	N/A	Leaves
**miRBase version**	14	16	16	19	20	20	20
**Reference**	*Arabidopsis thaliana* miRNAs from miRBase only	All mature Viridiplantae miRNas	All mature Viridiplantae miRNas	All mature Viridiplantae miRNas	All mature Viridiplantae miRNas	All mature Viridiplantae miRNas	All mature Viridiplantae miRNas
**Number of conserved miRNAs**	35	114	169	60	163	259 and 32	289
**Number of conserved miRNA families**	17*	56	34	27	32	13 and 7	30
**Number of Novel cassava-specific miRNAs**	0	12	0	821	17	N/A	39
**Number of Novel cassava-specific miRNA families**	N/A	N/A	N/A	N/A	N/A	N/A	29

* ESTs representing 7 of the families could not form secondary stem-loop structure and 3 families had MFEI <0.85

**Table 4:** miRNA families identified from miRBase V.20 in four previous studies compared with the miRNA families identified in this study.

						This Study		
Amiteye *et al*., 2010	Perez-Quintero *et al*., 2012	Balle-Taborda *et al*., 2013	Patanan *et al*., 2013	Xie *et al*., 2013	miR BAse V.20	EST	GSS	Deep-sequencing
*miR156*	miR1030	miR156	miR1446	MIR1446	miR1446	miR159	miR159	MIR156
*miR157*	miR156	miR157	miR156	MIR156	miR156	miR165	miR166	MIR157
miR159	miR159	miR159	miR159	MIR159	miR159	miR166	miR169	MIR159
**miR160**	miR160	miR160	miR160	MIR160	miR160	miR167	miR319	MIR160
*miR162*	miR164	miR164	miR162	MIR162	miR162	miR168	miR393	MIR162
**miR166**	miR166	miR166	miR164	MIR164	miR164	miR170	miR399	MIR164
miR167	miR167	miR167	miR166	MIR166	miR166	miR171	miR2275	MIR166
miR168	miR169	miR169	miR167	MIR167	miR167	miR394		MIR167
miR171	miR170	miR171	miR168	MIR168	miR168	miR399		MIR169
**miR172**	miR172	miR172	miR169	MIR169	miR169	miR408		MIR170
*miR390*	miR1863	miR2950	miR171	MIR171	miR171	miR482		MIR171
miR394	miR2111	miR319	miR172	MIR172	miR172	miR535		MIR172
*miR397*	miR390	miR390	miR2111	MIR2111	miR2111	miR2118		MIR2111
*miR398*	miR393	miR393	miR2275	MIR2275	miR2275			MIR2950
miR399	miR394	miR394	miR2950	MIR2950	miR2950			MIR319
miR408	miR395	miR395	miR319	MIR319	miR319			MIR390
*mi414*	miR396	miR396	miR390	MIR390	miR390			MIR393
	miR397	miR397	miR393	MIR393	miR393			MIR394
	miR403	miR398	miR394	MIR394	miR394			MIR395
	miR472	miR399	miR395	MIR395	miR395			MIR396
	miR477	miR408	miR396	MIR396	miR396			MIR397
	miR482	miR477	miR397	MIR397	miR397			MIR398
	miR535	miR482	miR399	MIR398	miR399			MIR399
	miR827	miR535	miR403	MIR399	miR403			MIR408
		miR6445	miR414	MIR403	miR408			MIR477
		miR827	miR473	MIR408	miR477			MIR482
		miR828	miR477	MIR477	miR482			MIR530
			miR482	MIR482	miR530			MIR535
			miR529	MIR530	miR535			MIR6445
			miR530	MIR535	miR827			MIR827
			miR535	MIR827	miR828			
			miR827	MIR828				
			miR828					
			miR845					

The study by Perez-Quintero *et al*. [55] addressed the role of miRNAs in the *Manihot esculenta*-*Xanthomonas axonopodis* pv. manihotis (*Xam*) interaction. NGS was used for analysing small RNA libraries from cassava leaf and stem tissue infected and uninfected with *Xam*. A full repertoire of cassava miRNAs was characterized, which included 114 individual conserved miRNAs belonging to 56 families and 12 novel cassava-specific miRNAs. This study used NGS to identify miRNAs in cassava and all available mature Viridiplantae miRNAs obtained from miRBase release (Version 16) were used as the reference when conducting the BLASTn search against the deep-sequencing reads [55] (Table 4). This was also the first study to identify cassava-specific miRNAs. A subsequent report by Patanun et al. [54], based on homology-based computational prediction, aimed to extend the cassava miRNA knowledge by using all reported plant miRNAs deposited in miRBase (V.16) to search against the cassava genome provided by Phytozome (http://www.phytozomenet/cassava) (Table 4). The cassava genome available at Phytozome was generated from the cassava cultivar AM5602. This study resulted in the identification of 169 individual conserved miRNAs belonging to 34 families in cassava (Table 4).

Ballen-Taborda et al. [57] used NGS and different bioinformatics methods to identify potential cassava miRNAs expressed in different tissues of the cassava cultivar TAI16 subjected to abiotic stress (heat and drought conditions), and the authors identified 821 novel miRNAs, but these were not submitted to miRBase. In comparison to Ballen-Taborda *et al*., a NGS study by Xie *et al*. [58], profiling miRNAs and target mRNA genes from cassava cv. SC124 plants that experienced severe and moderate chilling stresses, identified 163 individual conserved miRNAs belonging to 32 families and 17 cassava-specific miRNAs (Table 4). Our study combined a homology-based computational prediction approach using the publically available cassava EST and GSS databases at NCBI as well as a NGS of two different cassava landraces at three developmental stages (8, 10 and 15 weeks). Available Viridiplantae mature miRNAs from the updated miRBase V.21 were used as the reference for a BLASTn search for both approaches. In comparison to the previously mentioned studies above, NGS data from our study unveiled 289 individual miRNAs conserved in other plant species and 39 new previously unreported putative cassava-specific miRNAs (Table 4). Using the EST cassava database, 200 (77.2%) of the identified conserved individual miRNAs had not been reported in the above previous studies. Using the GSS cassava database, 22 (68.8%) of the identified individual conserved miRNAs had not been reported in the above previous studies, while NGS data revealed 230 (79.6%) of the individual conserved miRNAs had not been reported in the above previous studies. Additionally, we identified a miRNA family (miR2118) using the EST cassava database (underlined in Table 4) that not been reported in the above previous studies. For the novel cassava-specific miRNAs identified in this study, only one had been reported in a previous study. Therefore, 98.6% of the novel miRNAs identified in this study have not been previously reported. The miRNAs that were reported in the previous studies are bolded in S2–S5 Tables. From Tables 3 and 4, it is clear that the results of miRNA discovery studies in cassava varied depending on which miRNA identification method was used, which cultivar/landrace was studied, and not unexpectedly, if the plant underwent any biotic or abiotic stresses.

### Evolution of the identified conserved miRNA families in cassava

**Fig 8:**
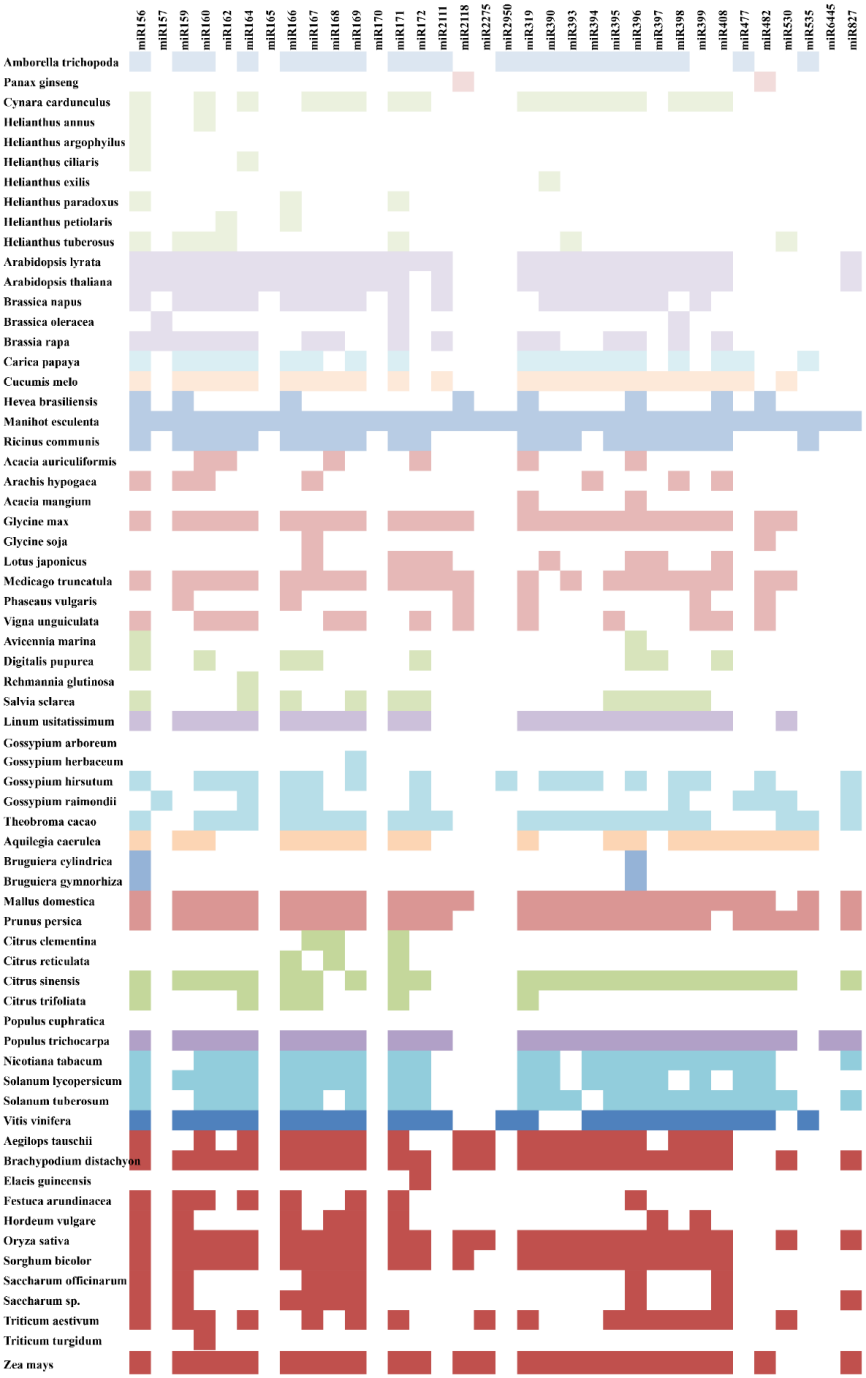
Evolutionary conservation of the thirty-four miRNA families identified in cassava within the plant species belonging to the Magnoliophyta Division (also known as Angiosperms) reported in miRBase (v. 21). Closely related species are shown in the same colour.

A small set of miRNAs has been detected in several major lineages of land plants [83]. Twenty-one miRNA families (miR156, miR159, miR160, miR162, miR164, miR166-169, miR171, miR172, miR319, miR390, miR393-399, and miR408) seem to be universally expressed among diverse plant species (Fig 8). A subset of these miRNA families is more ancient, because it is also present in gymnosperms, lycopods and bryophytes [83]. Eight miRNA families (miR156, miR159/319, miR160, miR166, miR171, miR408, miR390/391, and miR395) have been identified in the common ancestor of all embryophytes. The miR396 family is present in the common ancestor of all tracheophytes (vascular plants). The miR397 and miR398 families were acquired in the common ancestor of all spermatophytes (seed plants). Ten families (miR162, miR164, miR167, miR168, miR169, miR172, miR393, miR399 and miR827) are present in all angiosperm lineages (Fig 8). All of the above miRNA families were identified in cassava T200 and TME3 in this study.

*Populus trichocarpa* and *Ricinus communis* (castor bean) had 79.4% and 61.7% of miRNA families in common with cassava (Fig 8). This was expected as both these Euphorbiaceous species are closely related to *Manihot esculenta*, and *Populus trichocarpa* has been well studied in terms of its micronome, with 401 mature miRNA available on miRBase V.21. However, interestingly, *Populus euphratica* is also closely related to cassava but did not have a single miRNA family on common with cassava. This could be due to the lack of information available about its micronome as there are only 4 mature miRNAs available on miRBase (V. 21) for this plant species. *Glycine max* is a very well studied plant species with 639 mature miRNAs present on miRBase V.21 but while it is not closely related to cassava, interestingly, it shares 73.5% of the miRNA families with cassava.

At least nine families (miR441, miR444, miR818, miR821, miR1435, miR2118, miR2275 and miR582) likely arose in the monocot lineage [84]. However, in this study both the miR2118 and miR2275 were identified in cassava. MiR2118 has also been reported in 7 other dicot plant species in miRBase (V.21) [29], including the well-studied *Glycine max* species. MiR2275 has also been reported in cassava by Patanun *et al*. [54] and it has been accepted by miRBase as a true cassava miRNA. In rice both of these miRNA families have been implicated in secondary siRNA production. MiR2118 mediates the recruitment of 21 nt secondary siRNA-generating machinery and miR2275-targeted transcripts generate 24 nt siRNAs [85].

### Identification of targets

The prediction of miRNA targets is a significant step for validation of the newly identified cassava miRNAs. Most plant miRNAs have perfect or near perfect complementarity with their targets to regulate gene expression at post-transcriptional level [2, 86]. Based on this mechanism of miRNAs in plants, a homology search-based method was used for miRNA target prediction in cassava using psRNATarget server. The newly identified conserved and novel miRNAs in cassava were used as queries in the psRNATarget to predict the potential mRNA targets. Targets for conserved and novel miRNAs are detailed in S5 and S6 Tables, respectively.

#### Identification of targets for conserved miRNAs

**Fig 9:**
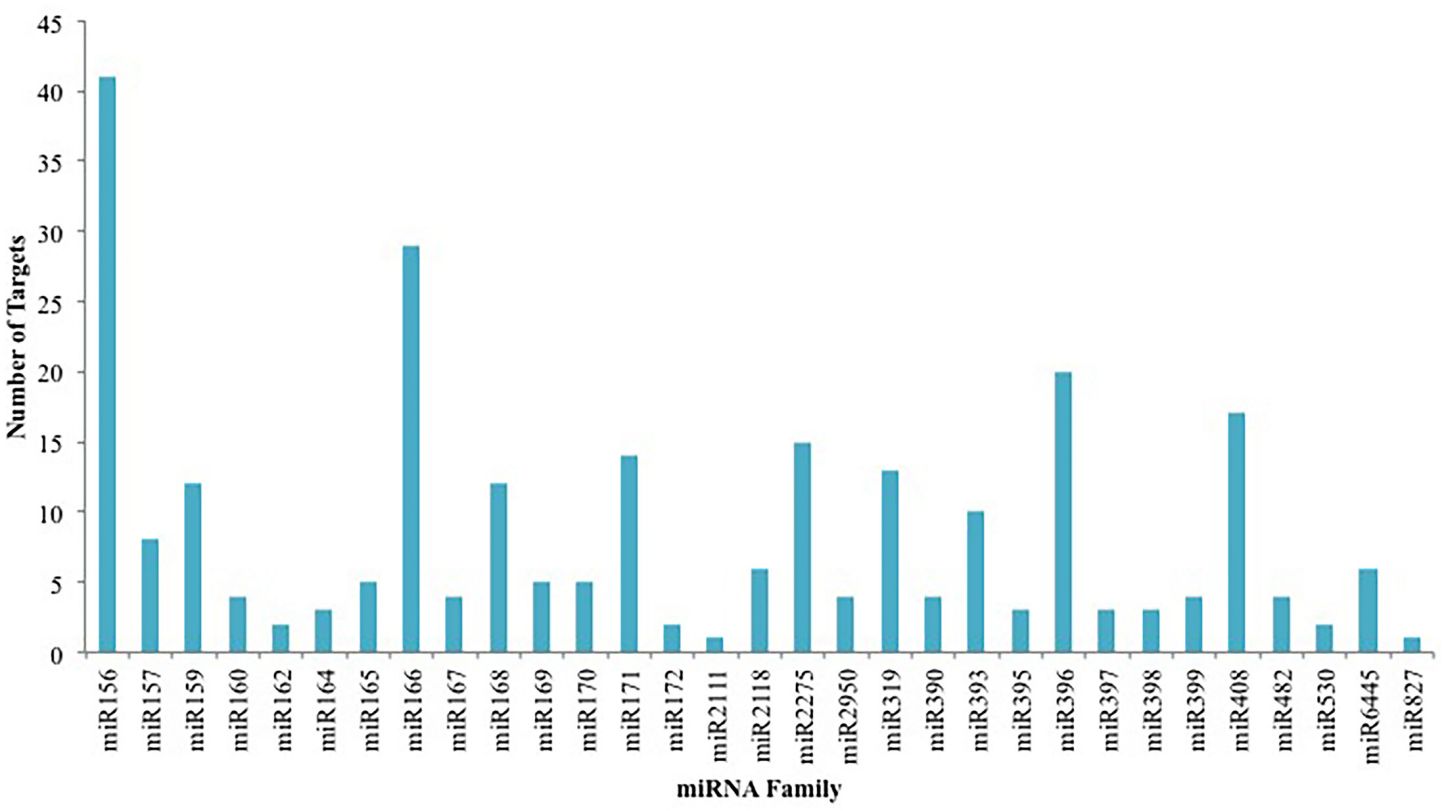
Number of targets predicted for each identified conserved miRNA in cassava.

Endogenous miRNAs act as negative regulators of gene expression by facilitating the cleavage of target mRNAs or by repressing their translation. The cleavage of target mRNAs seems to be a prime mode of gene regulation in plants [71]. In this study, 77.5% of the targets identified in cassava were repressed through cleavage while on 22.4% were repressed through translation. A total of 262 targets were identified for 32 of the conserved miRNA families identified in cassava using EST and GSS databases, and NGS data. The miR156 family had the most targets (41) followed by the miR166 family with 29 targets and miR396 with 20 targets (Fig 9). Only 111 of the targets were annotated with a known function. Transcription factors are important components in the transcription process and play an important role in a variety of biological functions, and therefore it was no surprise to observe 4 miRNA families (miR166, miR169, miR319 and miR408) in cassava were associated with 9 transcription factors. Several studies have indicated that miRNAs directly target the transcription factors that regulate plant development as well as specific genes that control various metabolic processes [87]. The miR169 family targets the CCAAT-binding transcription factor and the nuclear transcription factor Y subunit A-8. The CCAAT-binding transcription factor is a sequence-specific DNA binding transcription factor that is involved in double fertilization forming a zygote and endosperm. The nuclear transcription factor Y subunit A-8 stimulates the transcription of various genes by recognising and binding to a CCAAT motif in promoters [88]. The miR408 family targets a probable WRKY transcription factor 71 that interacts specifically with the W box (5’-(T) TGAC [CT]-3’), which is a frequently occurring elicitor-response cis-acting element [89].

The miR168 family, targeting AGO1, was also identified in cassava in this study. AGO1 is involved in RNA-mediated post-transcriptional gene silencing (PTGS). It is the main component of the RNA-induced silencing complex (RISC) that binds to a short guide RNA such as microRNA (miRNA) or small interfering RNA (siRNA) [90]. RISC then uses the mature miRNA or siRNA as a guide for slicer-directed cleavage of homologous mRNAs to repress gene expression. AGO1 mainly associates with miRNAs of 21 nt in length and preferentially recruits small RNAs with a 5' terminal uridine [74, 91]. It also associates with 22 nt miRNAs to trigger RDR6-dependent secondary siRNAs biogenesis [92]. This pathway amplifies silencing by using the target RNA as substrate to generate secondary siRNAs. It also binds to miR168, which targets its own mRNA for repression, establishing a homeostatic regulatory loop. AGO1 is involved in antiviral RNA silencing by contributing to viral RNA clearance [5]. This protein is also essential for multiple processes in development, including proper development of leaves and floral organs, and formation of axillary meristems. Like AGO10, it is required for stem cell function and organ polarity [25,93].

#### Identification of targets in cassava-specific novel miRNAs

**Fig 10:**
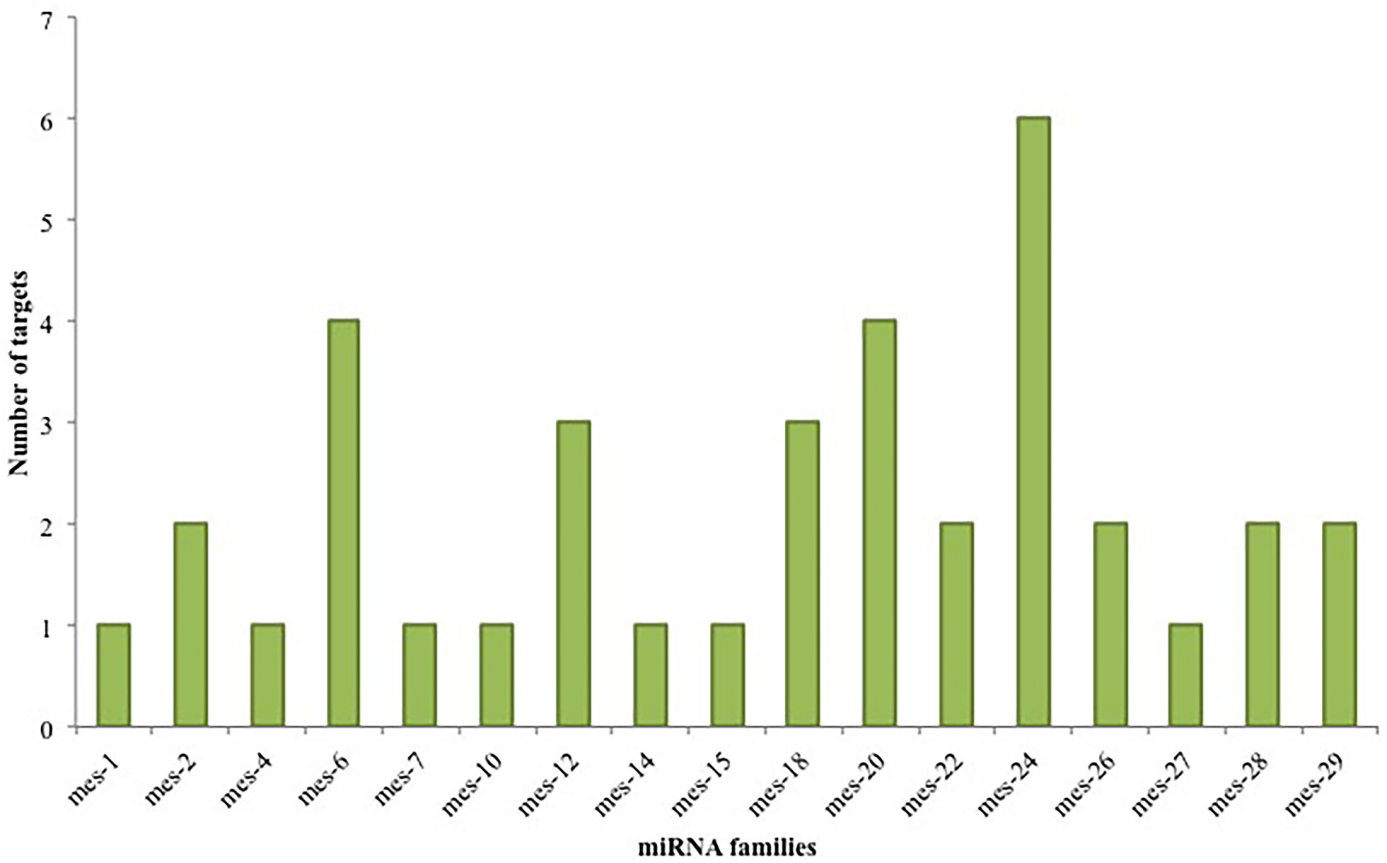
The number of targets predicted for the novel miRNA families identified in cassava.

In this study, 37 putative targets were predicted for 17 of the novel miRNA families identified in cassava (Fig 10). Mes-24 had the most targets, 6, followed by mes-20 and mes-6 with 4 targets, and mes-12 and 18 with 3 targets. The remaining novel miRNA families had 1 or 2 targets each.

Some of the targets identified for the novel miRNAs were known targets for some conserved miRNA families. An example is the mes-24 family with one of its targets being the Myb1 transcription factor. This transcription factor is a well-documented target for the miR159 family. An interesting target was the NBS-LRR Resistance protein RGH1, which is known to be involved in defense mechanisms in plants against pathogens. The cleavage of target mRNAs seems to be a prime mode of gene regulation in plants [71]. The majority of the novel miRNAs (70.5%) use cleavage to repress their targets, while 29.5% use translation as their repression mechanism.

#### GO annotations

**Fig 11:**
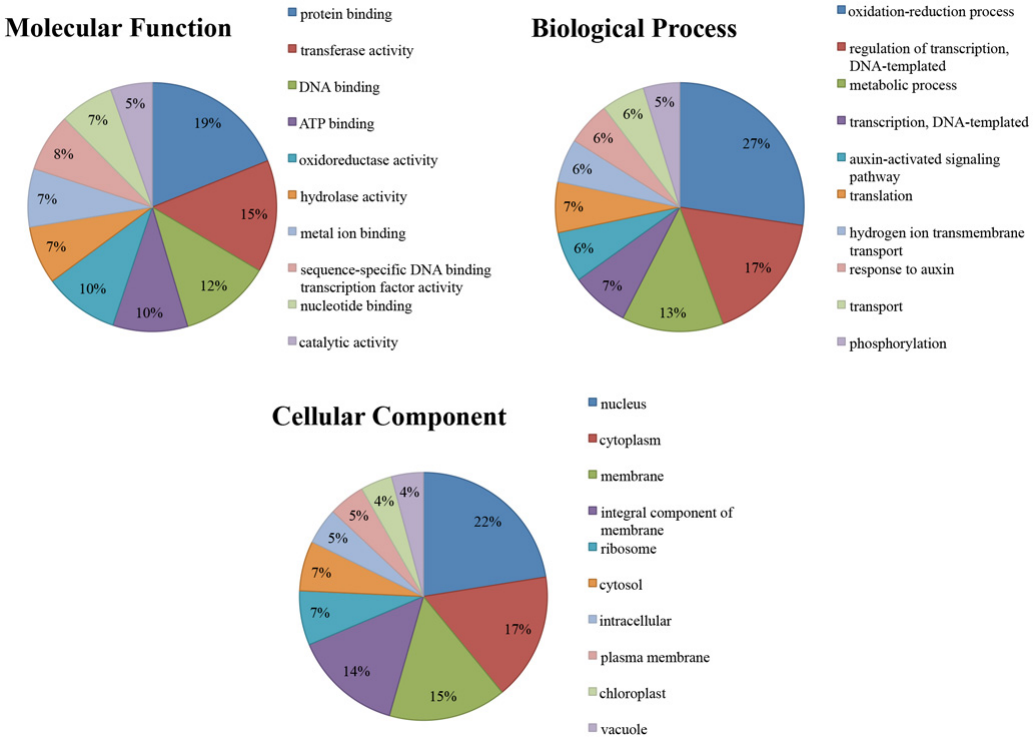
The top ten GO terms for the three GO categories for the targets identified for the conserved cassava miRNAs.

**Fig 12:**
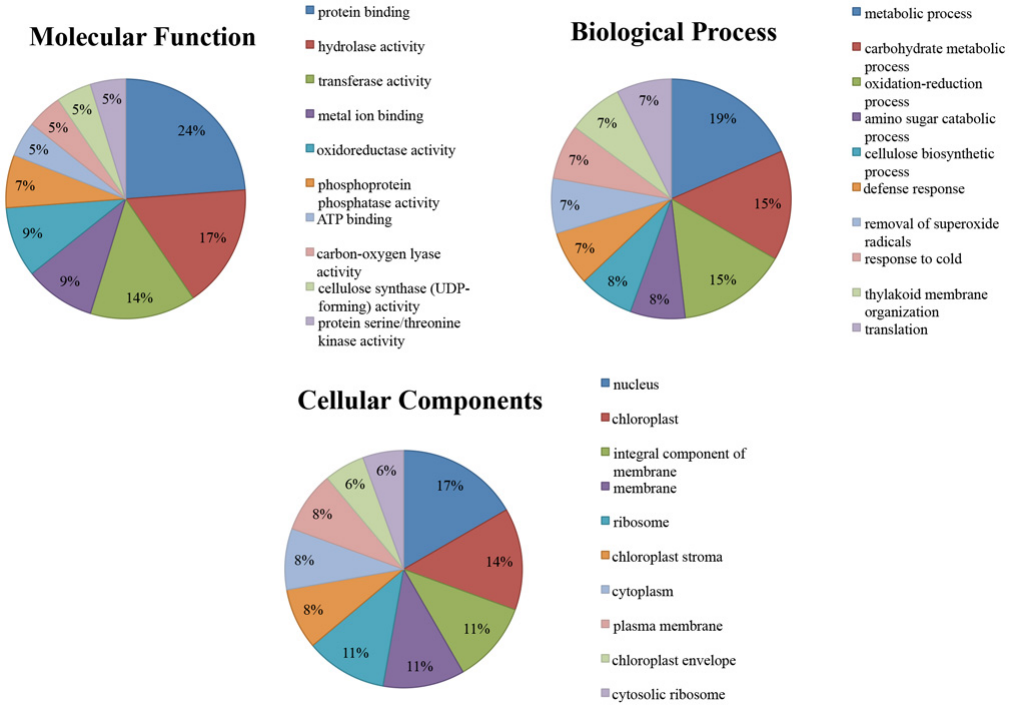
The top ten GO terms for the three GO categories for the targets identified for the novel cassava miRNAs.

To further understand the functions of the identified conserved and novel cassava miRNAs, the identified targets underwent GO term anaylsis. The results of this analysis are summarised in S7 and S8 Tables. GO term analysis allows the miRNA-gene regulatory network to be characterised in terms of molecular function, biological process and cellular component. The collective targets in T200 and TME3 from 3 developmental stages for the conserved miRNAs were involved in 101 molecular functions and 192 biological processes. The targets for the novel miRNAs were involved in 26 molecular functions and 37 biological processes. The top ten of the GO terms for each of the three GO categories for the targets identified for the conserved and novel miRNAs are represented in Figs 11 and 12. When comparing these results in the figures, the targets of the conserved and novel miRNAs had six molecular function terms, three biological processes terms and seven cellular componenets in common. These results suggest, not unexpectedly, that the cassava miRNAs are involved in various biological processes such as oxidation-reduction process, response to biotic and abiotic stresses, regulation of transcription and translation, transport, growth and development, and metabolism.

#### Secondary siRNA production

Many miRNAs, such as miR390 can direct their targets to generate phased trans-acting siRNAs (tasiRNAs) biogenesis, and the tasiRNAs then regulate other gene expression [46,94]. MiRNA-dependent transacting siRNAs (tasiRNAs), also known as phased siRNAs, are generated from noncoding TAS loci as well as protein-coding transcripts, and the secondary siRNAs can silence additional genes [46, 95] These miRNAs that trigger tasiRNAs are usually 22 nt in length. Notably, the miR390 identified in this study was not the typical 22 nt tasiRNA triggering 22 nt but was found to be 21 nt. This was also observed in a study by Montes *et al*. [96]. They found that the miR390 family was the only tasiRNA initiator to be present in all plant species studied and at high abundances but not expressed as a 22 nt. A member of the miR482 family identified in this study was also found to be 22 nt. Studies demonstrate that members of the miR482/2118 superfamily initiate large numbers of phased, secondary tasiRNA accumulation from plant *NB-LRR* class of resistance genes. For example in tomato, sequence diverse members of the miR482 family target large numbers of *NB-LRR* mRNAs, which in turn produce phased tasiRNAs [97], while in *Medicago truncatula* miR2118 causes large amounts of phased secondary tasiRNAs from *NB-LRR* mRNAs [98].

## Conclusions

In this study, 259 conserved miRNAs belonging to 32 families were identified using EST database, 32 conserved miRNAs belonging to 7 families identified using GSS database and 289 conserved miRNAs belonging to 30 families and 39 novel miRNAs belonging to 29 families were identified in T200 and TME3 landraces in deep-sequencing data. Also, 200 (77.2%) of the miRNAs in the EST library, 22 (68.8%) of the miRNAs identified in GSS, 230 (79.6%) of conserved miRNAs and 38 (98.6%) of the novel miRNAs identified in deep-sequencing data have not been previously reported in cassava. The mR2118 family identified in study has not been previously reported for cassava in other studies. However we could not experimentally detect this family using RT-PCR and this could be due to low expression levels or specificity of miRNA. Montes *et al*. [96] observed low miRNA abundance and conservation of mR2118. We observed that miRNA abundance increased as the conservation of the sequence increased and that our unique cassava specific miRNAs had an abundance of less than 5 RPM. By comparing miRNA identification in cassava in this study with others, it was demonstrated that the method used for miRNA identification, cultivar/landrace of cassava and environmental conditions can affect the miRNAs that are identified. It is notable that some divergence of miRNAs has taken place since cassava was introduced into West Africa in the 16^th^ century [99]. Differences between TME3 and T200 landraces can be hypothesised to have arisen from geographical separation and adaptation as T200 (history not known) is found in drier regions of southern Africa, while TME3 originates West Africa. Variations could have arisen from hybridizations with local wild *Manihot* species in different locations over the past few hundred years. While this research has unveiled some more important features of the cassava miRNAome, a large number of germplasm-specific cassava miRNAs of low abundance are likely not to have been detected.

## Materials and Methods

### Identification of miRNAs in cassava using EST and GSS database

#### Sequence databases

A total of 8524 known plant mature miRNA sequences were downloaded from miRBase database (http://www.mirbase.org/; Release 21: June 2014) [29]. The repeated miRNA sequences were removed to avoid redundant miRNAs and the remaining unique sequences were used as the reference set. The ESTs (86 310) and GSSs (77,569) available for cassava (collected from multiple germplasm) were downloaded from NCBI (http://www.ncbi.nlm.nih.gov).

#### Identification of putative conserved miRNAs in cassava

Two crucial filter conditions in EST and GSS analysis were used to identify conserved miRNAs: the conservation of mature miRNA sequences and the secondary structure of the pre-miRNA [34]. The mature sequences of all known plant miRNAs were used as a query for homologous searches against the cassava EST library and GSS database using BLAST search in CLC Main Workbench version 6.6.2. All cassava EST sequences with no more than 3 mismatches against the query sequences were saved. These initial candidate miRNA sequences predicted from the mature reference miRNAs were subjected for protein homology search at NCBI using BLASTx (http://www.ncbi.nlm.nih.gov/BLAST/) with default parameters and the protein coding sequences were removed.

#### Prediction of stem-loop secondary structures

The precursor sequences of the potential cassava sequences were subjected to hairpin secondary structure prediction using the RNA folding tool using default parameters in CLC Main Workbench version 6.6.2. The following criteria were used for selecting potential cassava pre-miRNAs [1, 49]: (1) Pre-miRNA could fold into a typical hairpin secondary structure and the mature miRNA was located in one stem; (2) the length of the pre-miRNA was no less than 50 nt; (3) pre-miRNA had a high minimal folding free energy (MFE) and MFE index (MFEI), which was calculated by MFEI = MFEx100/[length x (G+C%)], where length is the length of the RNA sequence and MFE is a negative folding free energy (-ΔG) [72]; (4) the maximum number of nucleotide mismatches between the mature miRNA and its opposite miRNA* sequence was six; [5] no loops or breaks in miRNA/miRNA* duplex was allowed.

### Identification of miRNAs in cassava using Next Generation Sequencing (NGS) data

#### Micropropagation and acclimatization of cassava

T200 and TME3 cassava landraces were micropropagated by way of nodal culture on Murashige and Skoog (MS) medium [100] supplemented with 20g.L-1 sucrose and 2g.L-1 Phytagel^™^ (Sigma Aldrich), pH 5.8. Explants for both landraces were grown under identical conditions, and were allowed to grow at 25°C under a 16 h photoperiod. At the appearance of roots (10 days), plantlets were transferred into Jiffy^®^ pellets which were placed on a tray that was covered with plastic film and placed in a insect free, temperature controlled growth chamber (28°C; 16 hour photoperiod). Slits were then gradually made in the plastic film to facilitate acclimatization of explants. Once acclimatised, the plantlets were potted with a 2:1 ratio of potting soil to vermiculite. The potted plants remained in the insect free, temperature-controlled growth chamber (28°C; 16 hour photoperiod). The average light intensity of the growth chamber was 3000 lux. The plants were watered every second day and once a month multifeed fertilizer was added to the plants, following manufacturer’s instructions. The newly developing upper most leaves were collected from the T200 and TME3 plants at 8, 10 and 15 weeks after the plantlets had been transferred to the Jiffy^®^ pellets.

#### RNA extraction, small RNA library preparation and sequencing

Total RNA extraction, using a modified high molecular weight polyethylene glycol (HMWPEG) protocol [101], was carried out on leaf tissue samples collected from T200 and TME3 at 8, 10 and 15 weeks. Six leaves from each plant in the replicate experiments were pooled to reduce variation. For each sample, 1g pooled leaf tissue was homogenised in liquid nitrogen and added to 5ml preheated (65°C) GHCL buffer (6.5 guanidium hydrochloride, 100mM Tris-HCl pH 8.0, 0.1M sodium acetate pH 5.5, 0.1M β-mercaptoethanol) and 0.1g HMW-PEG (Mr: 20 000, Sigma). The mixture was then pelleted by centrifugation (10000xg) for 10 minutes at 4°C. The supernatant was treated with 0.1ml 1M sodium citrate (pH 4.0), 0.2 ml 2M NaCl and 5 ml phenol:chloroform:isoamyl alcohol (PCI) (25:24:1). The mixture was then vortexed vigorously and again pelleted by centrifugation (10000 x g) for 10 minutes at 4°C. The supernatant was removed and RNA was precipitated by adding 5ml isopropanol (propan-2-ol). The mixture was thoroughly mixed and incubated at -20°C for 60 minutes and pelleted by centrifugation (10000 x g) for 25 minutes at 4°C. RNA pellets were washed with 5ml ice-cold 75% molecular grade ethanol. RNA Pellets were dried at 37°C for 5 minutes. The pellet was resuspended in 100μl preheated (55°C) RNase-free water and 1μl RNase inhibitor (Fermentas). Small RNAs were specifically filtered for using the mirVana^™^ miRNA isolation kit (Ambion Inc), following the manufacturer’s protocol. For cDNA library preparation, approximately 500 ng was used as input for the Illumina TruSeq Small RNA library preparation kit (Illumina, Inc.) and sequencing libraries were created according to the manufacturer’s protocol. Briefly, poly-A containing mRNA molecules were purified using poly-T oligo-attached magnetic beads. Following purification, the mRNA was fragmented and copied into first strand cDNA using random primers and reverse transcriptase. Second strand cDNA synthesis was then done using DNA Polymerase I and RNase H. The cDNA was then ligated to adapters and enriched with PCR to create the final cDNA library. The library was then pooled and sequenced on a HiSeq 2000 (Illumina, Inc.) instrument as per manufacturer’s instructions. Sequencing was performed up to 2 X 101 cycles. Next generating sequencing (NGS) was done using the Illumina HiSeq2000 platform at LGC Genomics in Berlin, Germany.

#### Small RNA sequencing analysis

Raw reads generated from the Illumina HiSeq2000 system for the 6 small RNA libraries were cleaned of sequence adapters using the fast-toolkit (http://hannonlab.cshl.edu/fastx_toolkit/), and low quality tags and small sequences (<15 nt long) were excluded. Quality analysis per cycle was performed for each library. To eliminate all other small non-coding RNAs, high quality trimmed sequences were mapped to rRNA, tRNA and snoRNAs sequences from Rfam (Version 12.0). The sequences that mapped completely and had an E-value <0.06 were removed from the libraries.

#### Identification of conserved miRNAs, isoforms, and novel miRNAs

In this study, CLC Genomics Workbench version 7.1 was used for data analyses. The FASTQ files containing the Illumina sequencing adapter clipped reads were imported into the CLC Genomics Workbench version 7.1 using the Import NGS option. The first step was to extract and count the small RNAs to create a small RNA sample that could be used for further analysis. The reads had previously had their adapters removed and trimmed. The maximum length of the small RNAs counted was set at 30 nt and the minimum length was 18 nt. The minimum sampling count was left as default, which was 1.

The small RNA samples produced when counting the tags was enriched by CLC Genomic Workbench by comparing the tag sequences with annotation resources such as miRBase. The integrated tool in the workbench was used to download miRBase. The downloaded version was the latest version, release 21, and was downloaded from ftp://mirbase.org/pub/mirbase/CURRENT/miRNA.dat.qz. The downloaded miRBase file contains all precursor sequences from the latest version of miRBase including annotations defining the mature miRNA regions. All plant species were selected from the list of species in miRBase. All settings were left as default except for the maximum mismatches, which was changed to 3.

The reads that mapped to the known miRNAs from miRBase with no more than 3 mismatches were then aligned to the cassava genome. Only the reads that mapped with no mismatches or gaps were considered to be potential miRNAs. In order to select for potential cassava pre-miRNAs, the region 250nt upstream and downstream from where the read mapped to the cassava genome was folded using the RNA folding tool in the CLC genomics workbench and was analysed using the secondary structure identification criteria mentioned previously [1, 49].

In order to predict novel miRNAs from cassava, the miRCat program in the UEA small RNA workbench was employed [98]. MiRCat identifies mature miRNAs and their precursors from a sRNA dataset and an input genome, AM5602 available at Phytozome (http://www.phytozomenet/cassava). The sRNA sequences are mapped to the input plant genome using PatMaN [102] and grouped into loci. In order to enrich for miRNA candidates, a number of criteria for the determination of a bona fide miRNA loci are applied by the software. In brief, the program searches for two-peak alignment patterns of sRNAs on one strand of the locus and evaluates the secondary structures of a series of putative precursor transcripts using the RNAfold [103] and randfold [50] programs. According to the recent criteria for annotating novel plant miRNAs, miRNA star (miRNA*) is one of the most important biogenesis proofs for the identification of a novel miRNA [51], and therefore only the identified novel miRNAs that had a corresponding miRNA* sequence identified were considered at potential cassava specific novel miRNAs.

### Experimental validation of selected miRNAs using reverse-transcription PCR

For the RT-PCR (reverse transcription) experimental validation, 7 conserved miRNAs and 6 novel miRNAs were randomly chosen from the predicted cassava miRNAs. The primers for the stem-loop sequences of these chosen miRNAs were designed using Integrated DNA technologies Primer Quest tool (www.idtdna.com/Primerquest/Home/index (S9 Table). Total RNA was extracted from cassava leaves using the Direct-zol^™^ RNA MiniPrep (Zymo Research), according to the manufacturer’s protocol. cDNA was synthesised using the RevertAid^™^ H minus First Strand cDNA synthesis Kit (Fermentas), according to the supplier’s protocol. One hundred ng cDNA was used as template for the PCR. The PCR was programmed as follows: initial denaturation at 95°C for 3 minutes followed by 34 cycles of denaturation at 95°C for 30 seconds, annealing at 59°C for 30 seconds, and extension at 72°C for 30 seconds and final elongation step at 72°C for 10 minutes. The PCR products were separated through 2% (w/v) agarose gel.

### Identification of targets and Gene Ontologies

Target genes were identified using psRNATarget server, an automated plant miRNA target prediction server available at plantgrn.noble.org.psRNATarget/ [104] using the *Manihot esculenta* (cassava), Unigene, DFCI Gene Index. The analysis parameters were set as default. Briefly, the following criteria were set for predicting the potential cassava miRNA target genes: (1) not more than four mismatches between identified miRNA and target mRNA; (2) no mismatches were allowed between positions 10th, 11th because this site was believed as a cleavage site; (3) one mismatch was allowed between position 2nd and 12th and up to three mismatches between position 12th and 25th; and (4) not more than two consecutive mismatches.

To better understand the functions of the newly identified potential targets, proteins were allocated gene ontology (GO) terms using Uniprot (www.uniprot.org).

## Supporting Information

S1 Table Summary of the characterisation of the identified conserved miRNAs for *cassava (Manihot esculenta)* using EST database. (XLSX)

S2 Table Summary of the characterisation of the identified conserved miRNAs for *cassava (Manihot esculenta)* using GSS database. (XLSX)

S3 Table Summary of the characterisation of the identified conserved miRNAs for *cassava (Manihot esculenta)* using deep-sequencing data. (XLSX)

S4 Table Summary of the characterisation of the identified novel miRNAs for cassava (*Manihot esculenta* Crantz) using deep sequencing data. (XLSX)

S5 Table Results from psRNATarget for the identified conserved miRNAs in cassava using EST and GSS databases, and deep-sequencing data. (XLSX)

S6 Table psRNATarget results for the novel miRNAs identified in cassava from deep-sequencing data. (XLSX)

S7 Table summary of the GO characterisation of the identified targets for the conserved miRNAs in terms of Biological process, Cellular components and Molecular function. (XLSX)

S8 Table summary of the GO characterisation of the identified targets for the novel miRNAs in terms of Biological process, Cellular components and Molecular function. (XLSX)

S9 Table Primer sequences used in this study for the RT-PCR experimental validation of the newly identified conserved and novel cassava miRNAs. (XLSX)
